# Supplementary figures and images for: Analysis of changes in intercellular communications in Alzheimer’s disease reveals conserved changes in glutamatergic transmission in mice and humans
Source: Sci Rep. 2025 Jul 19;15:26248. doi: 10.1038/s41598-025-10795-4 (PMC12276270; doi:10.1038/s41598-025-10795-4)

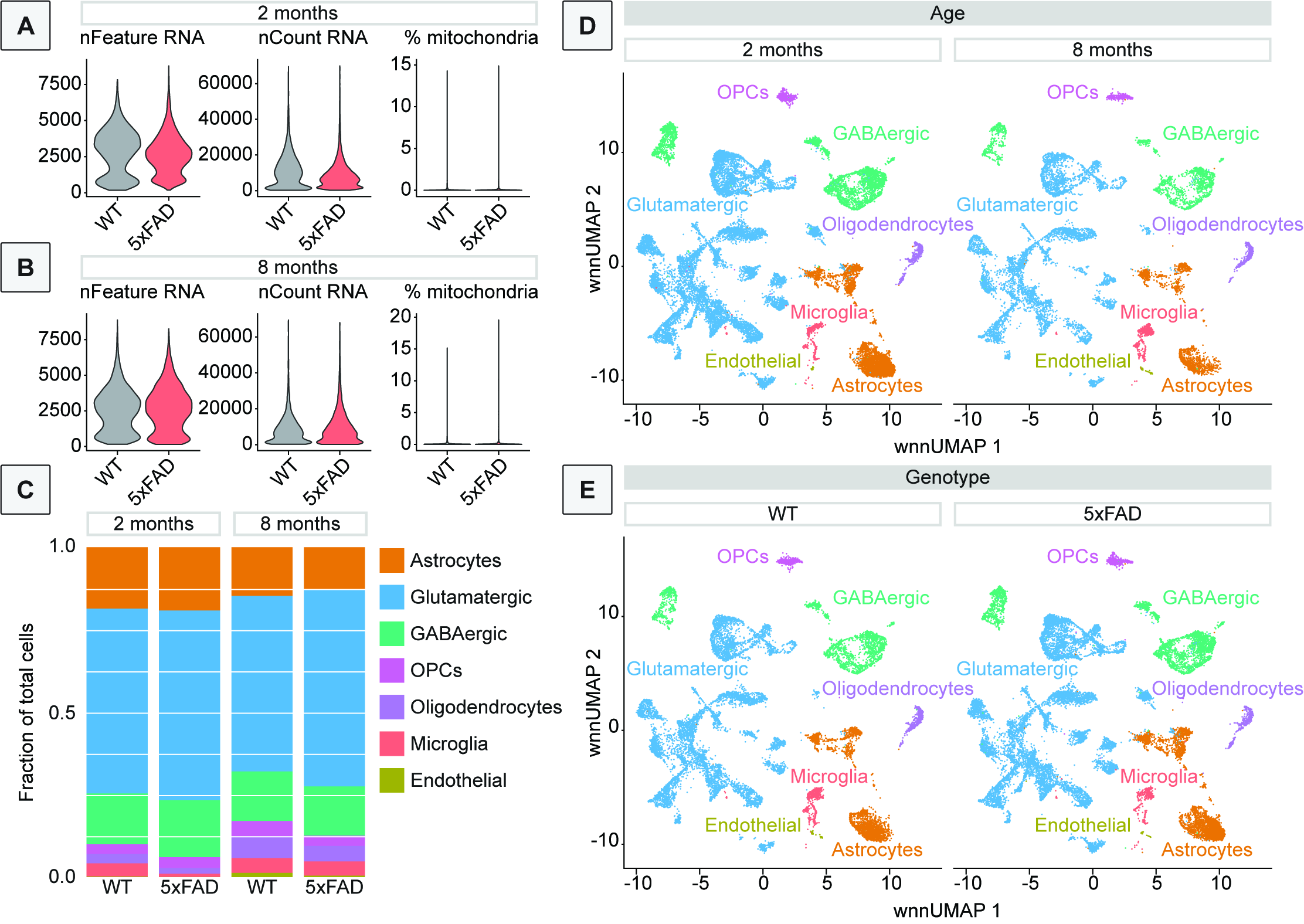

Supplement: Supplementary file 1 — Supplementary Material 1 [file 41598_2025_10795_MOESM1_ESM.tif]

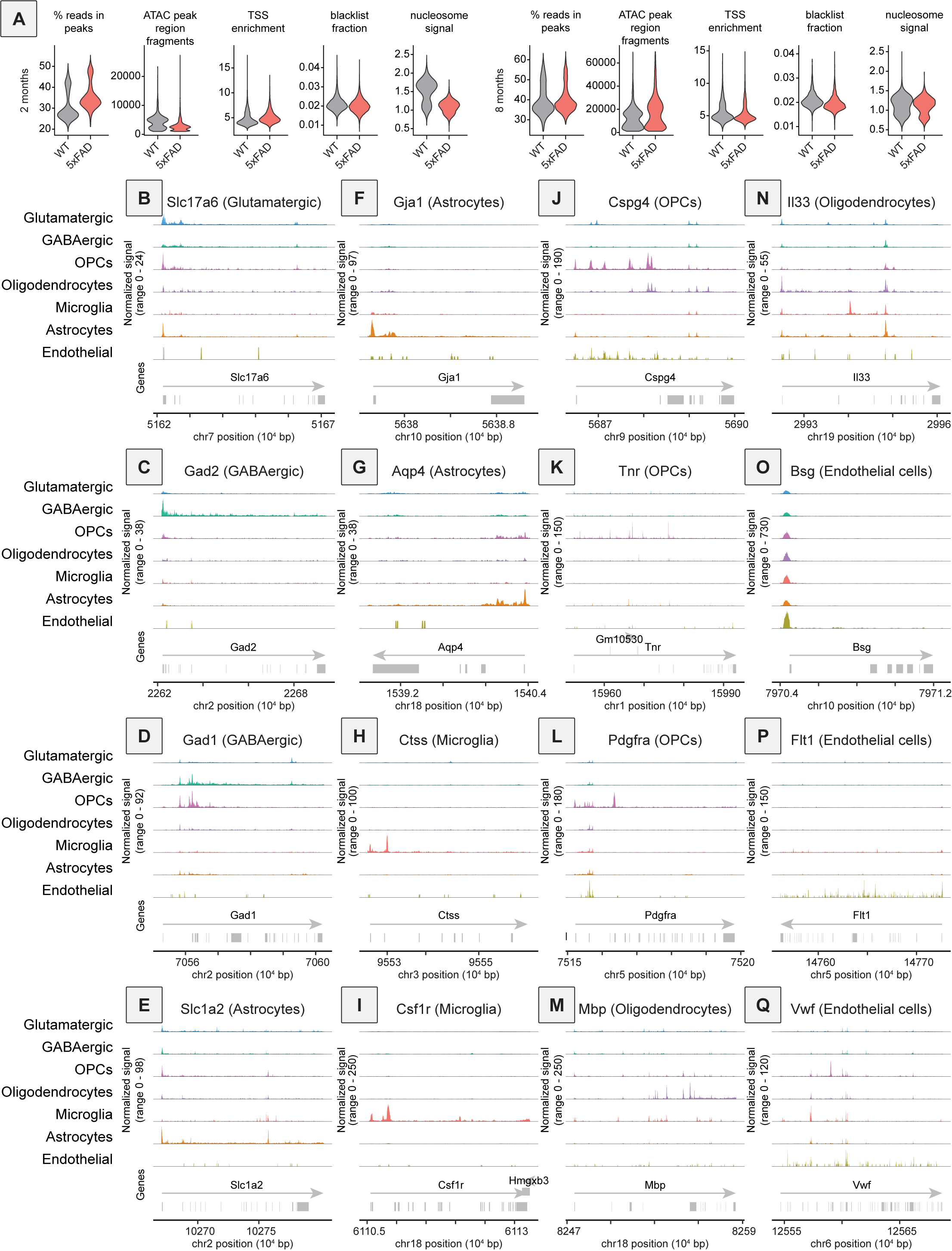

Supplement: Supplementary file 2 — Supplementary Material 2 [file 41598_2025_10795_MOESM2_ESM.tif]

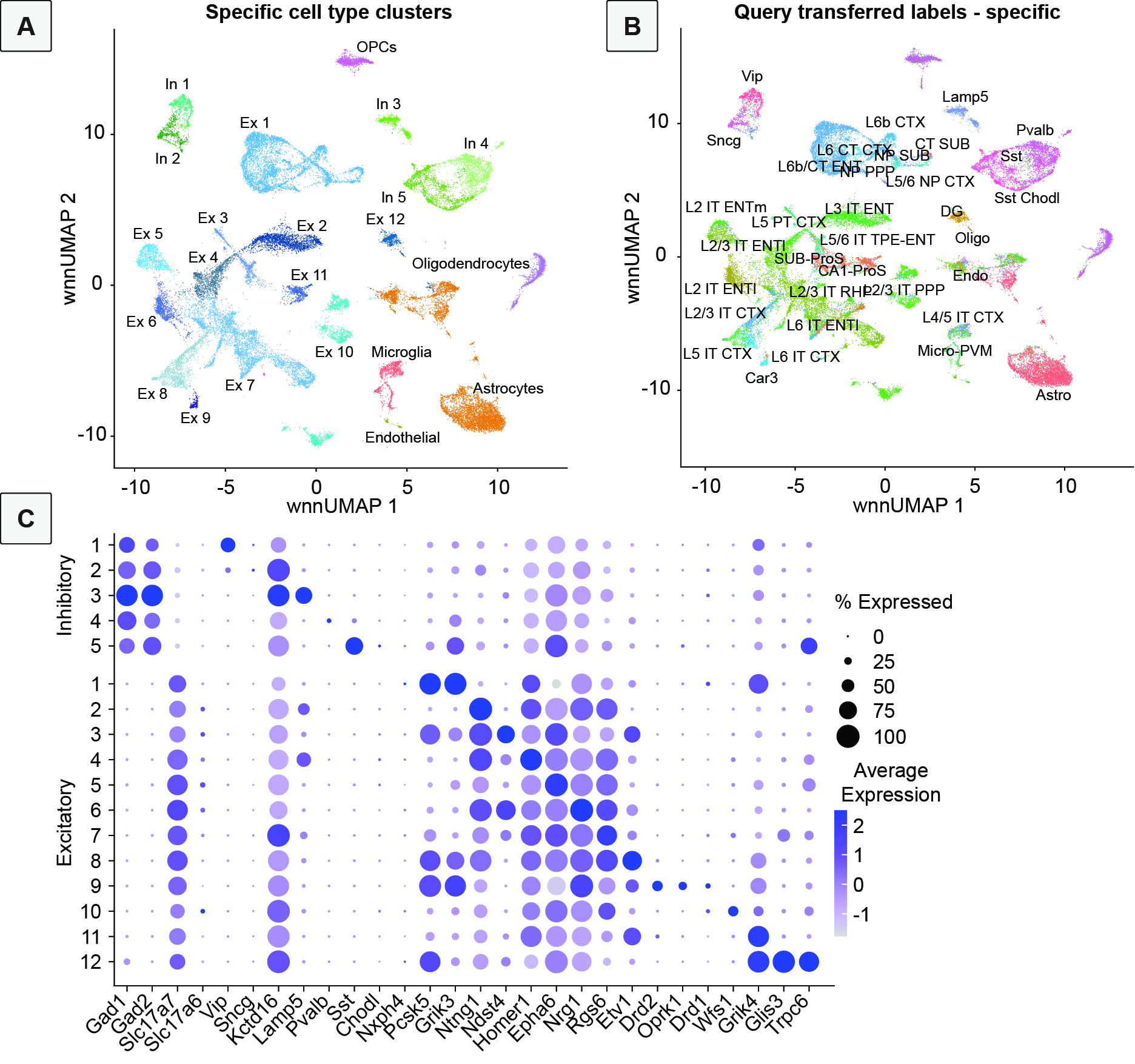

Supplement: Supplementary file 4 — Supplementary Material 4 [file 41598_2025_10795_MOESM4_ESM.tif]

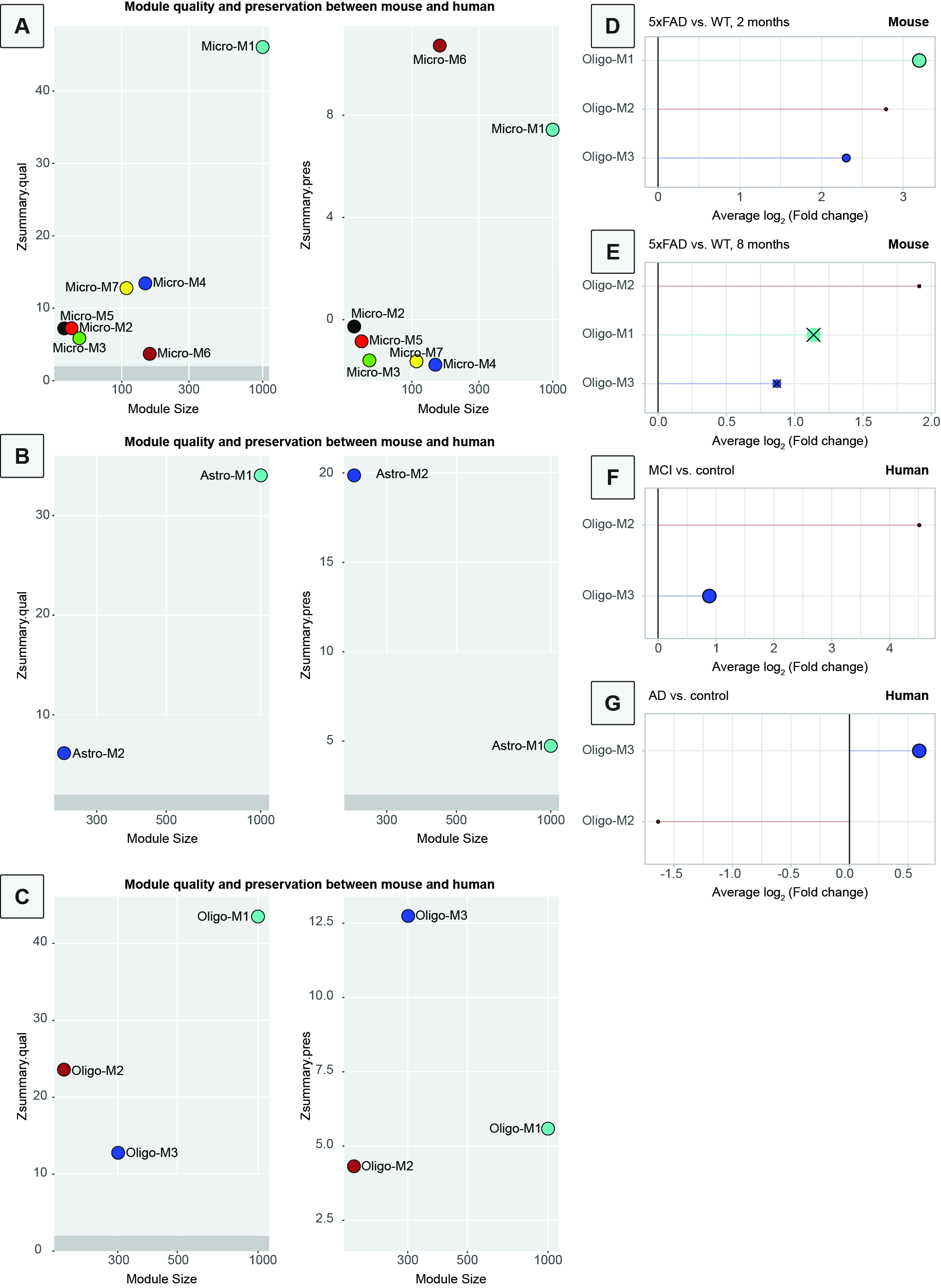

Supplement: Supplementary file 6 — Supplementary Material 6 [file 41598_2025_10795_MOESM6_ESM.tif]

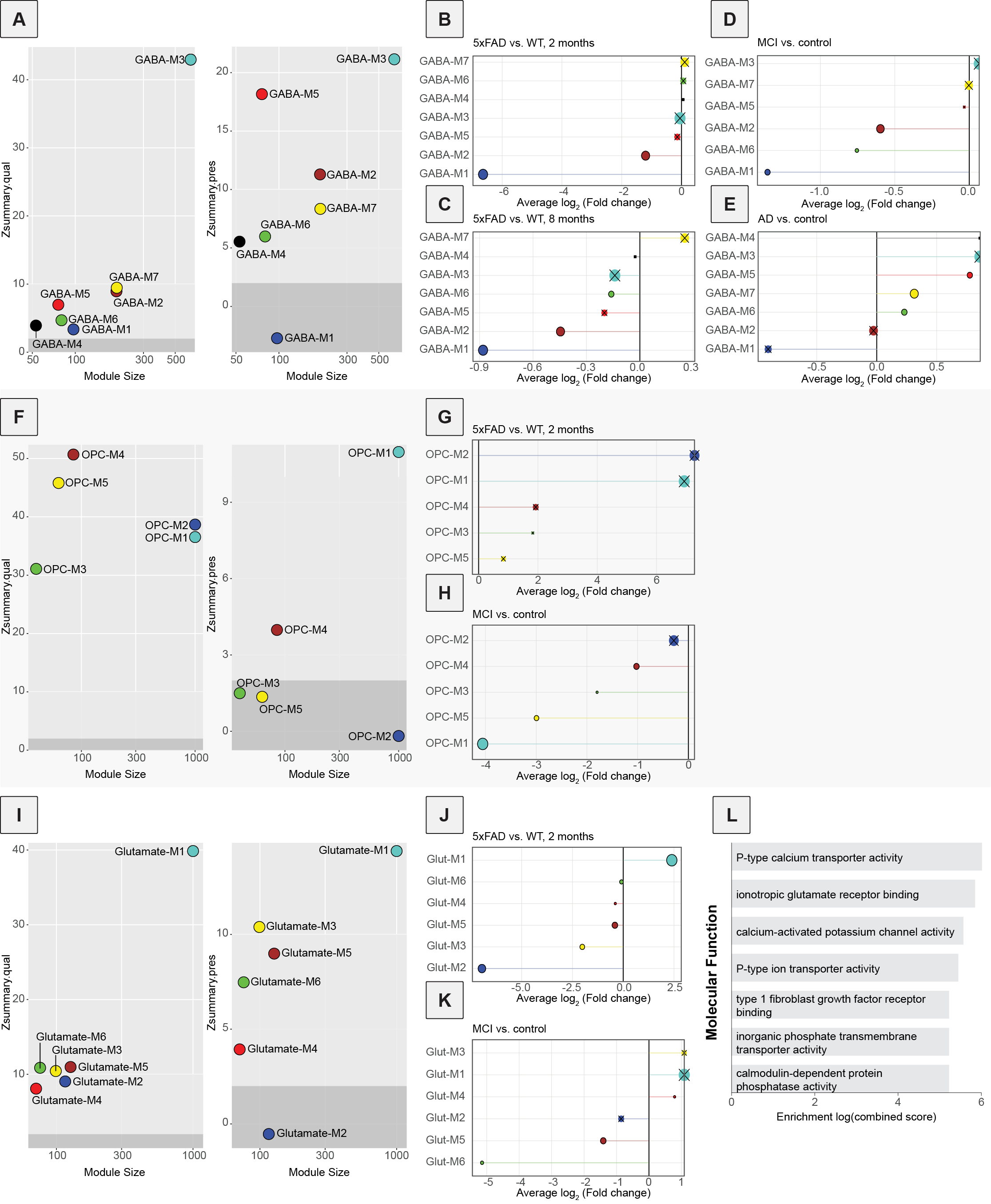

Supplement: Supplementary file 7 — Supplementary Material 7 [file 41598_2025_10795_MOESM7_ESM.tif]

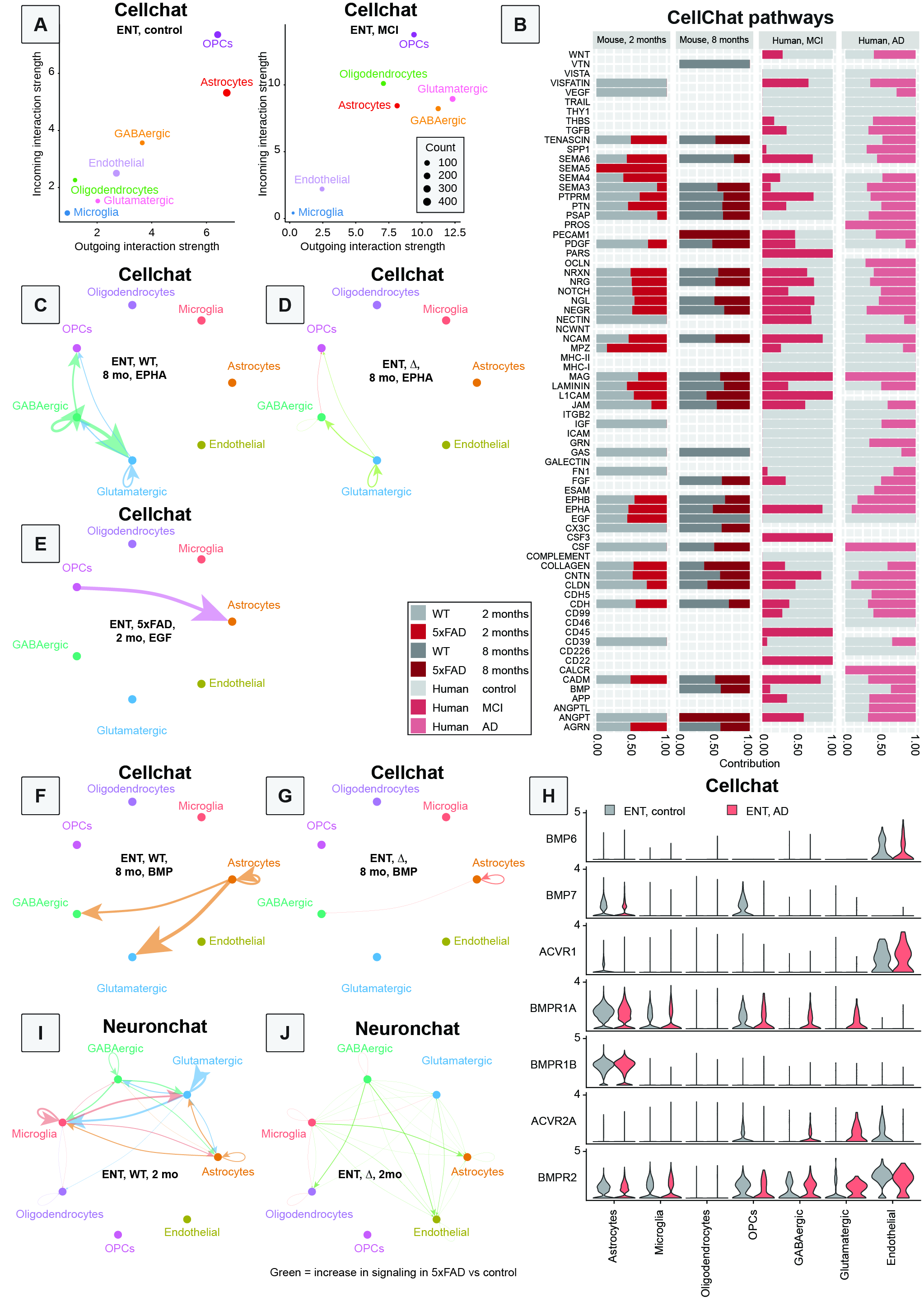

Supplement: Supplementary file 8 — Supplementary Material 8 [file 41598_2025_10795_MOESM8_ESM.tif]
