# Supplementary material for: Analysis of changes in intercellular communications in Alzheimer’s disease reveals conserved changes in glutamatergic transmission in mice and humans
Source: Sci Rep. 2025 Jul 19;15:26248. doi: 10.1038/s41598-025-10795-4 (PMC12276270; doi:10.1038/s41598-025-10795-4)
Supplement: Supplementary file 11 — Supplementary Material 11 [file 41598_2025_10795_MOESM11_ESM.docx]

**Supplementary Figure 1: Additional quality control metrics for sequencing data.**

**A**, Violin plots of QC metrics for snRNA-seq data after filtering out low-quality cells. Data from 5xFAD and control mice at 2 months of age are plotted. Metrics included from left to right are nFeature RNA (number of genes detected), nCount RNA (number of molecules detected), and percent mitochondria (percent mitochondrial genes).

**B**, Violin plots of QC metrics for snRNA-seq data after filtering out low-quality cells. Data from 5xFAD and control mice at 8 months of age are plotted.

**C**, Bar plots showing cell type proportions for 5xFAD and control mice in the sequencing data.

**D**, UMAP visualization of mouse sequencing data, split by age.

**E**, UMAP visualization of mouse sequencing data, split by genotype.

**Supplementary Figure 2: Quality control metrics for snATAC-seq data, and example gene accessibility plots for various cell types in mouse ENT.**

**A,** Violin plots of QC metrics for snATAC-seq data after filtering out undesirable cells. ATAC QC metrics shown for 5xFAD and control mice at 2 (left) and 8 (right) months of age. From left to right are percent reads in peaks, ATAC peak region fragments, transcription start site enrichment, fraction of reads in blacklist regions, and nucleosome signal.

**B,** Coverage plot for *Slc17a6*, a marker gene for glutamatergic neurons.

**C,** *Gad2*, a marker gene for GABAergic neurons.

**D,** *Gad1*, a marker gene for GABAergic neurons.

**E**, *Slc1a2*, a marker gene for astrocytes.

**F**, *Gja1*, a marker gene for astrocytes.

**G**, *Aqp4*, a marker gene for astrocytes.

**H**, *Ctss*, a marker gene for microglia.

**I**, *Csf1r*, a marker gene for microglia.

**J**, *Cspg4*, a marker gene for OPCs.

**K**, *Tnr*, a marker gene for OPCs.

**L**, *Pdgfra*, a marker gene for OPCs.

**M**, *Mbp*, a marker gene for oligodendrocytes.

**N**, *Il33*, a marker gene for oligodendrocytes.

**O**, *Bsg*, a marker gene for endothelial cells.

**P**, *Flt1*, a marker gene for endothelial cells.

**Q**, *Vwf*, a marker gene for endothelial cells.

**Supplementary Figure 3: Sub-clustering and further annotation of neurons in 5xFAD and control mouse data.**

**A**, UMAP visualization of mouse sequencing data with subclusters for different types of GABAergic, or inhibitory (In), neurons and glutamatergic, or excitatory (Ex), neurons.

**B**, UMAP visualization of mouse sequencing data annotated based on label transfer from data from the Allen Brain Atlas, including specific neuron types.

**C**, Dot plot showing gene expression differences and expression of known marker genes in the subclustered neuronal data as labeled in Figure S3A.

**Supplementary Figure 4: hdWGCNA results for microglia, astrocytes, and oligodendrocytes.**

**A**, zSummary metrics indicating module quality and preservation across species for modules found within the microglia cluster using hdWGCNA.

**B**, zSummary metrics indicating module quality and preservation across species for modules found within the astrocyte cluster using hdWGCNA.

**C**, zSummary metrics indicating module quality and preservation across species for modules found within the oligodendrocyte cluster using hdWGCNA.

**D**, DME analysis of oligodendrocyte modules in 2-month-old 5xFAD mice. All modules were upregulated in 5xFAD mice at 2 months of age. Circle size for panels D-G is indicative of the module’s size, with Oligo-M1 having the most genes, and Oligo-M2 having the least. For panels D-G, if a module is not significantly different between conditions, an ‘x’ indicates an adjusted p-value of > 0.05.

**E**, DME analysis of oligodendrocyte modules in 8-month-old 5xFAD mice. No modules were significantly different between genotypes at 8 months of age.

**F**, DME analysis of oligodendrocyte modules in postmortem tissue from people with MCI versus those without cognitive impairment.

**G**, DME analysis of oligodendrocyte modules in postmortem tissue from people with AD versus those without cognitive impairment.

**Supplementary Figure 5: Additional hdWGCNA results for GABAergic neurons, OPCs, and glutamatergic neurons.**

**A**, zSummary metrics indicating module quality and preservation across species for modules found within the GABAergic neuron cluster using hdWGCNA.

**B**, DME analysis of GABAergic neuron modules in 2-month-old 5xFAD mice. Circle size for panels B-E is indicative of the module’s size, with GABA-M3 having the most genes, and GABA-M4 having the least. For panels B-E, G-H, and J-K, if a module is not significantly different between conditions, an ‘x’ indicates an adjusted p-value of > 0.05.

**C**, DME analysis of GABAergic neuron modules in 8-month-old 5xFAD mice.

**D**, DME analysis of GABAergic neuron modules in MCI.

**E**, DME analysis of GABAergic neuron modules in AD.

**F**, zSummary metrics indicating module quality and preservation across species for modules found within the OPC cluster using hdWGCNA.

**G**, DME analysis of OPC modules in 2-month-old 5xFAD mice. Circle size for panels G-H is indicative of the module’s size, with OPC-M1 having the most genes, and OPC-M3 having the least.

**H**, DME analysis of OPC modules in MCI.

**I**, zSummary metrics indicating module quality and preservation across species for modules found within the glutamatergic neuron cluster using hdWGCNA.

**J**, DME analysis of glutamatergic neuron modules in 2-month-old 5xFAD mice. Circle size for panels J-K is indicative of the module’s size, with Glut-M1 having the most genes, and Glut-M4 having the least.

**K**, DME analysis of glutamatergic neuron modules in MCI.

**L**, GO analysis of genes in the GABA-M3 hdWGCNA module using the molecular function database.

#### **Supplementary Figure 6: Additional CellChat and NeuronChat results.**

**A**, Predicted outgoing and incoming cellular signaling with CellChat for each cell type in the ENT

of individuals without cognitive impairment (left) and predicted outgoing and incoming cellular signaling with CellChat for each cell type in the ENT of samples from donors with MCI.

**B**, Comparison of predicted signaling for all CellChat pathways in 5xFAD versus control mice at 2 and 8 months of age (left), and in individuals with MCI or AD versus those without cognitive impairment (right).

**C**, CellChat signaling inferred for EPHA in control mice at 8 months of age.

**D**, Differences in CellChat signaling inferred for EPHA in 5xFAD versus control mice at 8 months of age. An increase in signaling in 5xFAD vs control is indicated in green, and a decrease in signaling in red.

**E**, CellChat signaling inferred for EGF in 5xFAD mice at 2 months of age.

**F**, CellChat signaling inferred for BMP in control mice at 8 months of age.

**G**, Differences in CellChat signaling inferred for BMP in 5xFAD versus control mice at 8 months of age. Decreases in signaling are indicated in red.

**H**, Violin plots generated with CellChat showing expression of genes within the BMP signaling pathway in postmortem human tissue samples from people with AD and those without cognitive impairment. Included genes are BMP ligands, BMP receptors, and Activin receptors.

**I**, NeuronChat signaling inferred for all signaling pathways aggregated in control mice at 2 months of age.

**J**, Differences in NeuronChat signaling inferred for all signaling pathways in 5xFAD versus control mice at 2 months of age. An increase in signaling in 5xFAD vs control is indicated in green and a decrease in signaling in red.

**Supplementary Table 1: DGE results.**

**Supplementary Table 2: hdWGCNA modules.**

**Supplementary Table 3: DGE results for genes of interest and confidence intervals from jackknife resampling.**

**Supplementary Table 4: Demographic information from human brain donors used in this and published studies from which our data were derived.**
